# Activin Signaling in the Pathogenesis and Therapy of Neuropsychiatric Diseases

**DOI:** 10.3389/fnmol.2016.00032

**Published:** 2016-05-10

**Authors:** Andrea S. Link, Fang Zheng, Christian Alzheimer

**Affiliations:** Institute of Physiology and Pathophysiology, Friedrich-Alexander-Universität Erlangen-NürnbergErlangen, Germany

**Keywords:** activin, depression, anxiety disorders, drug craving, neurodegenerative disease

## Abstract

Activins are members of the transforming growth factor β (TGFβ) family and serve as multifunctional regulatory proteins in many tissues and organs. In the brain, activin A, which is formed by two disulfide-linked βA subunits, is recognized as the predominant player in activin signaling. Over the last years, considerable progress has been made in elucidating novel and unexpected functions of activin in the normal and diseased brain and in deciphering the underlying molecular mechanisms. Initially identified as a neurotrophic and protective factor during development and in several forms of acute injury, the scope of effects of activin A in the adult central nervous system (CNS) has been considerably broadened by now. Here, we will highlight recent findings that bear significance for a better understanding of the pathogenesis of various neuropsychiatric diseases and might hold promise for novel therapeutic strategies. While the basal level of activin A in the adult brain is low, significant short-term up-regulation occurs in response to increased neuronal activity. In fact, brief exposure to an enriched environment (EE) is already sufficient to considerably strengthen activin signaling. Enhancement of this pathway tunes the performance of glutamatergic and GABAergic synapses in a fashion that impacts on cognitive functions and affective behavior, counteracts death-inducing signals through extrasynaptic NMDA receptors (NMDARs), and stimulates adult neurogenesis in the hippocampus. We will discuss how impaired activin signaling is involved in anxiety disorders, depression, drug dependence, and neurodegenerative diseases such as Alzheimer’s and Parkinson’s, and how reinforcement of activin signaling might be exploited for therapeutic interventions.

## Introduction

Activins are members of the transforming growth factor β (TGFβ) family of growth and differentiation factors. They serve as multifunctional regulatory proteins in many tissues and organ systems, with particular emphasis on proliferation, differentiation, apoptosis, inflammation, immunoregulation and repair (Werner and Alzheimer, 2006; Hedger et al., 2011). Structurally, activins are homo- or heterodimeric proteins containing two disulfide-linked βA (encoded by the *Inhba* gene) and/or βB (encoded by the *Inhbb* gene) subunits. After proteolytic processing of their precursor proteins, activins are secreted as mature bioactive proteins. Activin A (βA/βA) is the most abundant and best characterized member of the activin family and plays the predominant role in activin signaling in the central nervous system (CNS). In the developing brain, activin A exerts distinct neurotrophic effects and is involved in proper cortical layering and corticostriatal wiring (Andreasson and Worley, 1995). Importantly, a recent study provided evidence that activin A supports neuronal differentiation of cortical neuronal progenitor cells (Rodríguez-Martinez et al., 2012). In the adult brain, activin A was originally identified as a neuroprotective factor in various forms of acute brain injury, including stroke (Wu et al., 1999; Tretter et al., 2000; Mukerji et al., 2009). In addition to its release from neurons, activin A can be also of glial origin, as demonstrated in a CNS demyelination model, where M2 microglia-derived activin A promotes oligodendrocyte differentiation and remyelination (Miron et al., 2013). The observation that the expression of activin A is strongly up-regulated in response to brain lesion and that both endogenous and recombinant activin A are capable of affording neuroprotection, led originally to the concept that, in the adult brain, activin A is a neuroprotective factor that is called to arms in emergency situations, when an initial damaging event, such as stroke, threatens to cause a massive neuronal loss.

Over the last decade, this somewhat narrow view on the role of activin has been substantially revised and extended. Our current understanding of the functions of activin A in the normal and diseased brain encompasses an astonishingly broad spectrum. On the physiological side of this range, activin regulates the daily operations of central synapses in a behaviorally relevant fashion. Major findings were that: (i) activin enhances cognitive performance by augmenting synaptic plasticity of excitatory (glutamatergic) synapses; and that (ii) by tuning inhibitory (GABAergic) synapses, activin sets the level of anxiety-like behavior. These findings are testimony to the impact of activin on mental faculties as well as on affective behavior. Since the effects of activin on central synapses of the healthy adult brain and their behavioral consequences have been reviewed elsewhere (Krieglstein et al., 2011), we will focus here on the role of activin signaling in neuropsychiatric disorders.

## Activin Signaling

**Figure 1:**
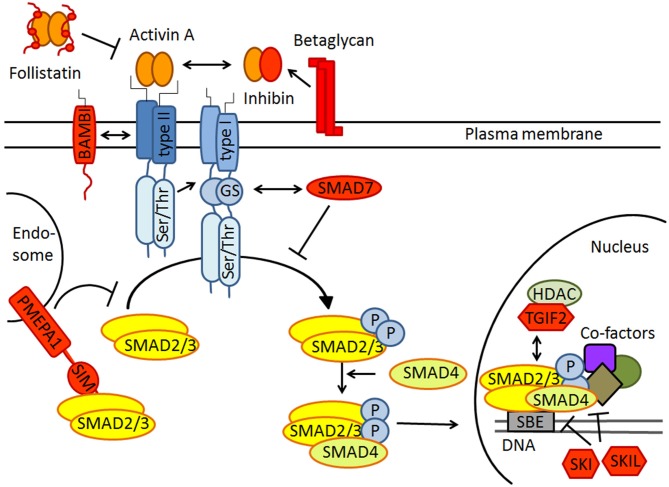
**Schematic drawing of canonical activin receptor signaling via SMAD2/3 proteins.** Note that SMAD2/3 signaling is strictly regulated by extracellular inhibitors like follistatin and inhibin, transmembrane antagonists such as BAMBI and PMEPA1, the cytoplasmatic inhibitory SMAD7 and several transcriptional repressors including SKI, SKIL and TGIF2 in the nucleus. All inhibitors are colored in red. For further explanation see text.

The activin signaling pathway is schematically depicted in Figure 1. Activins signal through heteromeric complexes of type II (ActRIIA, ActRIIB) and type I receptors (predominantly ActRIB, but also ActRIA and ActRIC). Type II receptors bind activin and recruit type I receptors, which then phosphorylate the intracellular signaling proteins SMAD2/3. These assemble with SMAD4, translocate to the nucleus, and bind to specific target genes to modulate their expression (Chen et al., 2006; Xia and Schneyer, 2009). It is important to stress that the strength and duration of activin signaling are tightly controlled at several nodes along the signaling pathway (Choi and Han, 2011). In the schematic drawing of Figure 1, negative regulators of activin signaling are colored in red. Well-known inhibitors are the secreted proteins follistatin and inhibin, which preclude activin receptor binding through direct physical interaction and receptor competition, respectively. Betaglycan promotes the interaction between inhibin and ActRII, thereby counteracting activin binding and signaling (Hedger et al., 2011). In the cell membrane, the pseudoreceptor BAMBI (BMP and activin membrane-bound inhibitor) inhibits transmission in a ligand-independent manner. PMEPA1 (prostate transmembrane protein, androgen induced 1) directly binds to SMAD2/3 and thus prevents their phosphorylation by activated type I receptors. Notably, PMEPA1 was recently identified as an activin target gene in the brain (Link et al., 2015), suggesting that it might serve as a feed-back inhibitor of activin signaling. The intracellular inhibitor SMAD7 competitively blocks SMAD2/3 binding sites at type I receptors. In the nucleus, the transcriptional co-factors SKI, SKIL and TGIF2 act as transcriptional repressors. In addition to this canonical signaling pathway, activins can also activate other pathways, including mitogen-activated protein kinase (MAPK) signaling (Moustakas and Heldin, 2005).

## Activin Signaling in Conditioned Fear, Anxiety, and Drug Dependence

Although anxiety and fear both describe forms of affective behavior, which occur in anticipation of threatening stimuli, be they real or envisioned, it is conceptually helpful to make a distinction between these terms. We will refer to fear responses as conditioned behaviors elicited by a particular cue or environmental context. By contrast, anxiety manifests itself as a form of behavioral inhibition in conditions of conflict, when, for example, a novel, but potentially threatening object or environment requires a choice between approach vs. avoidance. Conditioned fear has been linked to long-term synaptic plasticity of glutamatergic synapses in the amygdala (Pape and Pare, 2010), with the contextual information provided by the hippocampus. Anxiety-like behavior appears to be primarily controlled by the (ventral) hippocampus (Bannerman et al., 2004). As will be detailed in the next section, activin signaling impinges on both, conditioned fear and anxiety-like behavior.

### Conditioned Fear

Ageta et al. (2010) generated forebrain-specific activin- or follistatin-transgenic mice in which the transgene expression was regulated by the Tet-OFF system, allowing temporally controlled over-expression of activin or its inhibitor follistatin. If already over-expressed during training in a contextual fear-conditioning test, follistatin reduced freezing behavior tested 24 h later. When follistatin or activin were over-expressed *after* training, reconsolidation of fear memory was weakened or strengthened, respectively (Ageta et al., 2010; Ageta and Tsuchida, 2011). The finding that inhibition of activin signaling during memory retrieval is capable of suppressing previously consolidated fear memories is of particular translational interest, because it advances activin signaling as a putative therapeutic target to extinguish traumatic memories and fear-laden stimuli in human anxiety disorders such as posttraumatic stress disorder (PTSD) and phobias.

How does activin signaling interfere with acquisition, retrieval and extinction of conditioned fear? In view of the dependence of contextual fear conditioning on synaptic plasticity in amygdalar and hippocampal circuits, the known effects of activin signaling on hippocampal long-term potentiation (LTP) offer a plausible clue. Andreasson and Worley (1995) were the first to demonstrate up-regulation of activin βA mRNA in the dentate gyrus (DG) *in vivo* in response to LTP-inducing electrical stimulation of the perforant path. Underscoring the functionality of this up-regulation, later studies found that forebrain-specific disruption of activin receptor signaling and over-expression of follistatin impaired LTP in area CA1 and DG (Muller et al., 2006; Ageta et al., 2010). Mechanistically, activin augments LTP by targeting several of its essential mechanisms, including NMDA receptor (NMDAR) currents and spine density and morphology (Muller et al., 2006; Shoji-Kasai et al., 2007; Kurisaki et al., 2008; Hasegawa et al., 2014).

### Anxiety, Alcohol and GABA_A_ Receptors

Transgenic mice expressing a dominant-negative ActRIB in forebrain neurons display a low-anxiety phenotype in typical conflict paradigms in which the innate drive to explore a novel environment is opposed by the tendency to stay in safe surroundings (Zheng et al., 2009). The decrease in anxiety-like behavior in these mice has been linked to concomitant alterations in GABAergic inhibition, based on a number of conspicuous parallels: (i) Tonic inhibition by extrasynaptic GABA_A_ receptors (GABA_A_Rs), which is thought to exert anxiolytic effects (Brickley and Mody, 2012; Whissell et al., 2015), is enhanced in the mutant mice; (ii) GABA_A_Rs of the mutant mice show substantially less sensitivity to diazepam, concomitant with a loss of the anxiolytic drug effect at the behavioral level; and (iii) GABA_B_R function is augmented in the transgenic mice, consistent with reports that positive GABA_B_R modulators reduce anxiety-like behavior (Zheng et al., 2009).

Given that disruption of activin receptor signaling reduces allosteric modulation of GABA_A_Rs by diazepam, it came as a surprise that ethanol potentiation of GABA_A_R-mediated inhibitory synaptic transmission was enhanced in the same transgenic mice (Zheng et al., 2015). As a consequence, low concentrations of ethanol (≤30 mM) are now capable of potentiating the inhibitory effects of synaptic GABA_A_Rs. Loss of neuronal activin receptor signaling renders not only GABA_A_Rs more sensitive to ethanol, but also produces significantly stronger sedation at the behavioral level (Zheng et al., 2015). These data suggest that activin regulates behavioral effects of ethanol by controlling ethanol potentiation of GABA_A_R function, arguably the pre-eminent target of ethanol in the brain. At the molecular level, activin appears to adjust ethanol sensitivity of GABA_A_Rs through a non-canonical, i.e., SMAD2/3-independent signaling pathway involving PKCε (Zheng et al., 2015). Notably, this PKC isoform has been previously shown to regulate the responsiveness of GABA_A_Rs to ethanol (Hodge et al., 1999).

### Cocaine Craving

While activin is involved in the sedating, but not in the reinforcing effects of ethanol (Zheng et al., 2015), a recent study implicated activin signaling in maladaptive processes leading to drug craving and relapse after cocaine withdrawal (Gancarz et al., 2015). After 7 days, but not after 1 day of withdrawal following cocaine self-administration in rats, phosphorylation of SMAD3 was specifically increased in the shell of the nucleus accumbens (NAc). Intra-accumbal microinjection of activin A increased drug self-administration, whereas viral over-expression of a dominant-negative mutant of SMAD3 (dnSMAD3) in the NAc had the opposite effect. Moreover, dnSMAD3 abrogated the increase in the density of dendritic spines on medium spiny neurons in the NAc, which typically accompanies drug-induced reinstatement of cocaine self-administration (Gancarz et al., 2015). Such structural plasticity following withdrawal is considered essential for the endurance of cocaine-seeking behavior, and activin signaling appears to be a driving force behind this rewiring process. As pointed out earlier, changes in spine morphology have been also proposed as a mechanism through which activin promotes hippocampal LTP and memory formation. This leaves us with the intriguing idea that, depending on whether it is recruited endogenously (e.g., by learning processes in the hippocampus) or hijacked exogenously (e.g., by drugs of abuse in the NAc), activin signaling can operate both ways, increasing cognitive performance as well as instituting drug-seeking behavior and relapse.

## Activin Signaling and Depression

### Activin Signaling as a Target in Antidepressant Treatment

Given the co-morbidity of mood and anxiety disorders, it should not come unexpected that activin signaling has also been assigned a role in depression. Specifically, several proteins of the activin signaling pathway were found to be up-regulated in rodent brain after chronic administration of clinically used antidepressants. Chronic and subchronic paroxetine produced an increased expression of *Inhba* mRNA and of ActRIA mRNA in circumscribed regions of mouse hippocampus (Ganea et al., 2012). While not influencing *Inhba* expression, chronic fluoxetine and desipramine did increase SMAD2 phosphorylation in rat frontal cortex (Dow et al., 2005). Furthermore, genetic polymorphism in *TGFBR3* which codes for betaglycan, a functional inhibitor of activin binding and signaling (v.s.), has been associated with the clinical response to antidepressant drugs (Ganea et al., 2012). Electroconvulsive therapy (ECT), the second mainstay of antidepressant treatment, engenders a strong and rapid (within 2 h) increase of *Inhba* mRNA in hippocampus, amygdala and several neocortical areas, followed by enhanced SMAD2 phosphorylation, indicating subsequent activation of the canonical activin signaling pathway in this rodent model of ECT (Dow et al., 2005; Link et al., 2015). Compared to other members of the TGFβ family, which also signal via SMAD2/3, only *Inhba* expression was significantly increased, strongly suggesting that ECT gives rise exclusively to activin A protein (Link et al., 2015). Taken by themselves, these findings do not necessarily indicate a role of activin as a mediator of the therapeutic effects of antidepressant drugs or ECT. It was therefore reassuring that stereotactic infusion of recombinant activin A into rat and mouse hippocampus produced antidepressant-like effects in the forced swim test, a behavioral model of depression (Dow et al., 2005; Ganea et al., 2012). Notably, the antidepressant-like effects were only observed when activin A was injected into the DG, but not when injected into the CA1 region or into the amygdala (Dow et al., 2005; Ganea et al., 2012).

### Activin, Depression, Adult Neurogenesis and Epigenetic Modification

The DG clearly emerges as the essential brain region linking activin signaling to its antidepressant effects. In view of the (not undisputed) neurogenic hypothesis of depression, which posits that adult-generated neurons of the DG are required for balanced mood and antidepressant efficacy (Eisch and Petrik, 2012), an obvious question is whether the therapeutic effects of activin can be causally related to increased adult neurogenesis. Whereas it remains controversial whether the basal level of endogenous activin in the intact adult brain is sufficient to influence baseline neurogenesis, administration of activin A to the normal hippocampus did enhance neural stem/precursor cell proliferation (Ageta et al., 2008; Abdipranoto-Cowley et al., 2009). These findings would be consistent with the concept that the dramatic up-regulation of activin by antidepressant treatment contributes to disease remission by promoting hippocampal neurogenesis.

**Figure 2:**
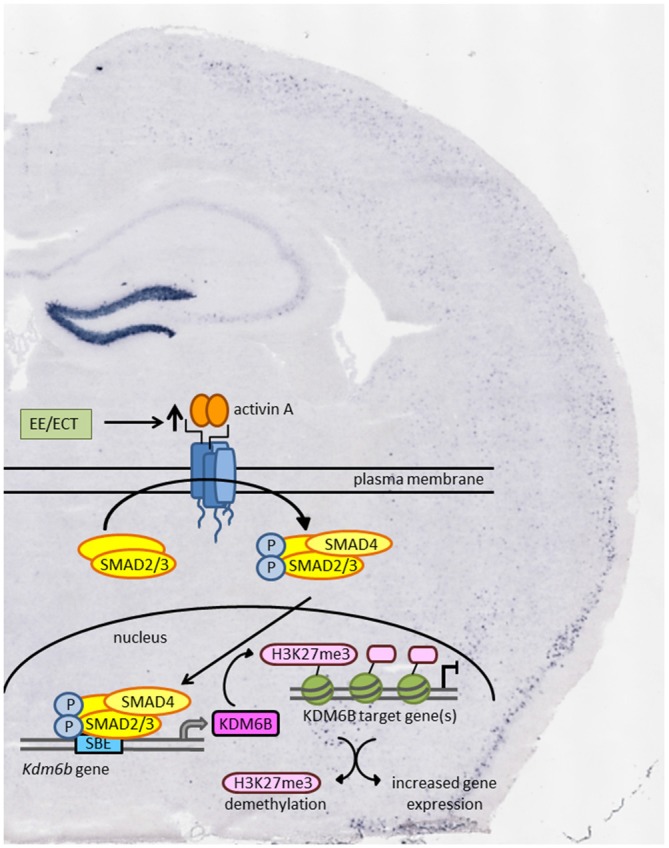
**Schematic model of how environmental enrichment (EE) and electroconvulsive therapy (ECT) may produce epigenetic modification through enhanced activin signaling and transcription of the activin target gene *Kdm6b*.** KDM6B acts as a histone demethylase thereby promoting gene expression of transcriptionally repressed genes. Background: *in situ* hybridization of coronal mouse brain section showing pronounced increase in *Inhba* mRNA level in dentate gyrus (DG) 2 h after ECT-like electrical stimulation.

With the identification of the histone demethylase KDM6B as the product of an activin target gene in the brain, epigenetic regulation might emerge as a new effect of activin signaling that could contribute to its antidepressant effects (Link et al., 2015). According to this hypothesis, enhanced activin signaling following ECT or antidepressant pharmacotherapy would lead to chromatin remodeling and increased transcriptional activity as schematically depicted in Figure 2.

Finally, activin A might also serve as an endogenous antidepressant in its own right, independent of its up-regulation by antidepressant treatment. Strong support for this notion comes from experiments with mice placed into an enriched environment (EE). Compared to mice housed in standard cages, EE exposure produced a marked up-regulation of *Inhba* mRNA in the DG and the CA3 region of the hippocampus, accompanied by increased SMAD2/3 phosphorylation (Link et al., 2015). These findings suggest that the mood-elevating and cognition-enhancing effects attributed to EE might be mediated, at least in part, by enhanced activin signaling.

## Activin and Neurodegenerative Diseases

In an excitotoxic rodent model of Huntington’s disease, intrastriatal administration of recombinant activin A for 1 week rescued striatal interneurons and projection neurons from death (Hughes et al., 1999). Administration of recombinant activin A also protected dopaminergic midbrain neurons against MPP^+^-induced cell death *in vitro* (Krieglstein et al., 1995) and in the 6-OHDA mouse model of Parkinson’s disease (Stayte et al., 2015). While these studies suggest that activin signaling might be targeted to fight off gradual neuronal demise in chronic neurodegenerative disease, the underlying mechanism had been elusive until recently. A widely held concept posits that a proper balance between signals transmitted by synaptic vs. extrasynaptic NMDARs is an essential determinant of neuronal integrity (Hardingham and Bading, 2010). Activation of *synaptic* NMDARs triggers synaptic plasticity and fosters genetic programs for stress resilience and survival. By contrast, activation of *extrasynaptic* NMDARs can incur lethal consequences for neurons, as it initiates pro-apoptotic pathways. Lau et al. (2015) have now revealed an intriguing interplay between brain-derived neurotrophic factor (BDNF) and activin signaling that is instrumental to shift the delicate balance between extrasynaptic and synaptic NMDARs in favor of the latter, thereby strengthening neuronal health and long-term survival. In brief, this survival-promoting scheme comprises the following sequence of events. Activity-dependent increase in *Bdnf* transcription or administration of exogenous BDNF promotes activation of synaptic NMDARs, thereby inducing nuclear calcium signaling, which in turn activates transcription of *Inhba*. Secreted activin A then reduces potentially neurotoxic Ca^2+^ influx through extrasynaptic NMDARs, thereby counteracting pro-death pathways (Lau et al., 2015). Based on such a scenario, it seems plausible to assume that any perturbation of the neuroprotective interaction between BDNF and activin A should render neurons more vulnerable to excitotoxic damage and might thus represent an important pathogenetic factor in protracted neurodegenerative diseases such as Huntington’s, Parkinson’s or Alzheimer’s.

## Outlook

The characterization of *Inhba* as a BDNF-induced gene and the identification of the sequential and interdependent activation of BNDF and activin signaling as a health-promoting mechanism offer an intriguing new perspective on possibly shared functions of these two factors that might well go beyond their involvement in combating neuronal loss in neurodegenerative diseases. A quick review of the multiple roles of BDNF in the adult brain reveals a broad spectrum of effects that show a striking overlap with those of activin. Like activin, BDNF has been implicated in activity-dependent regulation of synaptic plasticity (Minichiello, 2009; Edelmann et al., 2014). In fact, the effects of BDNF bear a strong resemblance with those of activin in that both factors alter key morphological and electrophysiological features of glutamatergic synapses, thereby providing a double underpinning for neuronal processes intimately involved in learning and memory. Moreover, several lines of evidence show that BDNF, like activin, crucially modulates the strength of GABAergic inhibition (Gottmann et al., 2009). In addition to their involvement in anxiety disorders (Liu et al., 2004; Martinowich et al., 2007) and drug addiction (Li and Wolf, 2015), activin and BDNF appear to be endowed with antidepressant efficacy (Martinowich et al., 2007). Both signaling pathways are stimulated by EE and, much more so, by ECT, as well as by antidepressant drug treatment (Nibuya et al., 1995; Link et al., 2015). With BDNF and activin being up-regulated strongest in the DG, both factors were found to stimulate adult neurogenesis, which is thought to support remission of depression (Martinowich et al., 2007). Thus, several lines of evidence indicate that BDNF and activin synergistically co-regulate neuronal responses, suggesting essential cross-talk of their signaling pathways.

In contrast to BDNF, which has long since taken center stage as a master regulator of various neuronal properties and functions during development and in adulthood, the multiple roles of activin are only gradually being uncovered. It seems that, in many aspects, activin and BDNF are two players with very similar overall targets, which join forces in neuroprotection, synaptic plasticity and neuropsychiatric diseases. The commonalities and differences in the strategies these two factors pursue to achieve these goals and the extent to which the underlying mechanisms are complementary and interdependent should be exciting topics for future research.

## Author Contributions

The manuscript was written by CA, with contributions of ASL and FZ. Schematic drawings were designed by ASL.

## Conflict of Interest Statement

The authors declare that the research was conducted in the absence of any commercial or financial relationships that could be construed as a potential conflict of interest.
